# Translation and Validation of a Brief Health Literacy Instrument for School-Age Children in a Danish Context

**DOI:** 10.3928/24748307-20220106-01

**Published:** 2022-01

**Authors:** Ane H. Bonde, Nanna W. Stjernqvist, Charlotte D. Klinker, Helle T. Maindal, Olli Paakkari, Peter Elsborg

## Abstract

Low health literacy (HL) is associated with adverse health behaviors and poor health, and brief, high-quality instruments for measuring HL in children are scarce. The Health Literacy for School-Aged Children (HLSAC) instrument is a 10-item theory-based and internationally validated tool for measuring HL. The purpose of this study was to translate and validate the HLSAC instrument among Danish school-aged children. The instrument was translated into Danish by a standardized forward-backward translation process, and then pre-tested for face validity with 61 pupils from four schools. Thereafter, the instrument was tested among 805 pupils in grades 6 to 7 (age 11–14 years) from 15 schools. When HL was modeled as one latent factor with all 10 items loading on this factor, the confirmative factor analysis showed standardized factor loadings from 0.52 to 0.75 (*p* < .001) and an excellent model fit. The association between HL and food intake as a health behavior example (*p* < .001, r^2^ = .027) indicates the predictive validity of the instrument. The internal consistency was high (Cronbach's alpha = 0.86). Thus, a valid and reliable version of the HLSAC instrument is available in Danish for future surveys to monitor HL and guide health promotion targeting children and adolescents. **[*HLRP: Health Literacy Research and Practice*. 2022;6(1):e25–e29.]**

Low health literacy (HL) is a predictor of adverse health behaviors and poor health status in adults (Berkman et al., 2011). From a health promotion perspective, improving HL among younger people is crucial, as evidence suggests that health behavior tracks from childhood into adulthood (Patton et al., 2016). However, high-quality HL instruments for children and youth are scarce (Guo et al., 2018), and few instruments are comprehensive, generic, and brief enough for population surveys (Okan et al., 2018). One such tool is the Health Literacy in School-Aged Children (HLSAC) instrument (Paakkari et al., 2016). It has 10 items and is based on five theoretically derived components of HL: theoretical knowledge, practical knowledge, critical thinking, self-awareness, and citizenship (Paakkari & Paakkari, 2012). The instrument was validated among Finnish children in grades 7 and 9 (age 13–15 years), and later applied in four European countries, showing that mean HL values are comparable across countries (Paakkari, Torppa, Boberova, et al., 2019). No validated HL instrument targeting children exists in Danish. Therefore, the purpose of this study was to translate and validate the HLSAC instrument among Danish children, and thereby enabling a bridge between high quality research and best practice. The target group was pupils in grades 6 to 7; grade 7 as in the original study, and grade 6 to add new knowledge by testing the instrument in an age group younger by 1 year.

## Methods

The validation process was conducted in three phases following recommendations for questionnaire translation (Eremenco et al., 2005) and scale validation (Boateng et al., 2018).

First, two translators independently translated the instrument into Danish, and two other translators back translated the Danish version into English. The primary focus was on achieving conceptual equivalence between the English and the Danish versions, with semantic equivalence being secondary. Three issues arose that required special attention from the expert committee that reviewed the process: which Danish words to use for “surrounding natural environment” (item 6) and “figure out” (item 9), and how best to translate the response categories to secure a clear continuum from option 1 to 4 (**Table 1**).

**Table 1:**
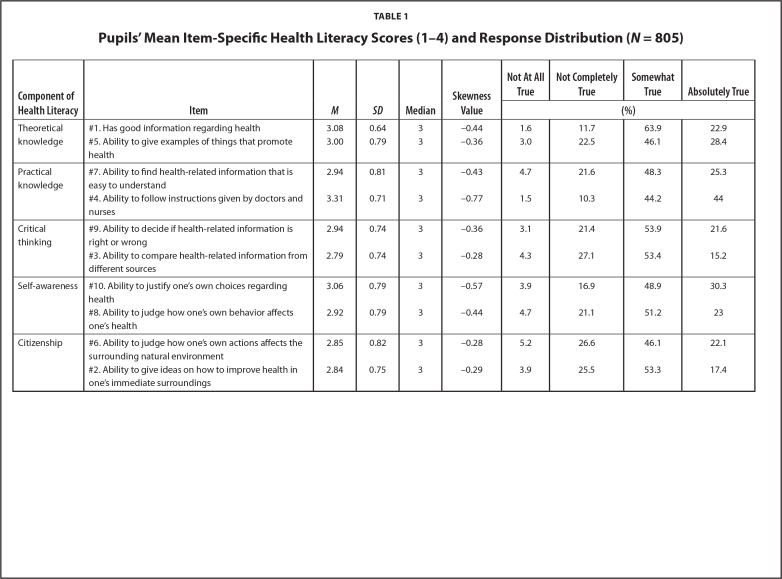
Pupils' Mean Item-Specific Health Literacy Scores (1–4) and Response Distribution (*N *= 805)

**Component of Health Literacy**	**Item**	***M***	***SD***	**Median**	**Skewness Value**	**Not At All True**	**Not Completely True**	**Somewhat True**	**Absolutely True**

						**(%)**			

Theoretical knowledge	#1. Has good information regarding health	3.08	0.64	3	−0.44	1.6	11.7	63.9	22.9
	#5. Ability to give examples of things that promote health	3.00	0.79	3	−0.36	3.0	22.5	46.1	28.4

Practical knowledge	#7. Ability to find health-related information that is easy to understand	2.94	0.81	3	−0.43	4.7	21.6	48.3	25.3
	#4. Ability to follow instructions given by doctors and nurses	3.31	0.71	3	−0.77	1.5	10.3	44.2	44

Critical thinking	#9. Ability to decide if health-related information is right or wrong	2.94	0.74	3	−0.36	3.1	21.4	53.9	21.6
	#3. Ability to compare health-related information from different sources	2.79	0.74	3	−0.28	4.3	27.1	53.4	15.2

Self-awareness	#10. Ability to justify one's own choices regarding health	3.06	0.79	3	−0.57	3.9	16.9	48.9	30.3
	#8. Ability to judge how one's own behavior affects one's health	2.92	0.79	3	−0.44	4.7	21.1	51.2	23

Citizenship	#6. Ability to judge how one's own actions affects the surrounding natural environment	2.85	0.82	3	−0.28	5.2	26.6	46.1	22.1
	#2. Ability to give ideas on how to improve health in one's immediate surroundings	2.84	0.75	3	−0.29	3.9	25.5	53.3	17.4

Next, the translated instrument was pre-tested for face validity involving 61 pupils in four schools. Four focus group interviews on the pupils' understanding of the items were conducted among 20 pupils. Further, the instrument was distributed to two classes (41 pupils) and comments were collected from the pupils. The face validity assessment resulted in a change in item 5, where the Danish word for “promote” (as in “promote health”) was replaced with the word for “improve.” For complete Danish translation, see **Table A**.

**Table A:**
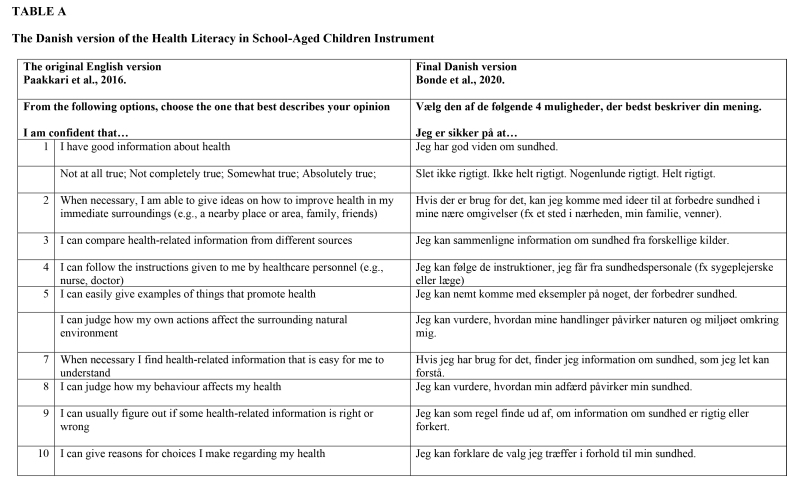
The Danish version of the Health Literacy in School-Aged Children Instrument

**The original English version Paakkari et al., 2016.**		**Final Danish version Bonde et al., 2020.**

**From the following options, choose the one that best describes your opinion**		**Vælg den af de følgende 4 muligheder, der bedst beskriver din mening.**
**I am confident that…**		**Jeg er sikker på at…**

1	I have good information about health	Jeg har god viden om sundhed.

	Not at all true; Not completely true; Somewhat true; Absolutely true;	Slet ikke rigtigt. Ikke helt rigtigt. Nogenlunde rigtigt. Helt rigtigt.

2	When necessary, I am able to give ideas on how to improve health in my immediate surroundings (e.g., a nearby place or area, family, friends)	Hvis der er brug for det, kan jeg komme med ideer til at forbedre sundhed i mine nære omgivelser (fx et sted i nærheden, min familie, venner).

3	I can compare health-related information from different sources	Jeg kan sammenligne information om sundhed fra forskellige kilder.

4	I can follow the instructions given to me by healthcare personnel (e.g., nurse, doctor)	Jeg kan følge de instruktioner, jeg får fra sundhedspersonale (fx sygeplejerske eller læge)

5	I can easily give examples of things that promote health	Jeg kan nemt komme med eksempler på noget, der forbedrer sundhed.

	I can judge how my own actions affect the surrounding natural environment	Jeg kan vurdere, hvordan mine handlinger påvirker naturen og miljøet omkring mig.

7	When necessary I find health-related information that is easy for me to understand	Hvis jeg har brug for det, finder jeg information om sundhed, som jeg let kan forstå.

8	I can judge how my behaviour affects my health	Jeg kan vurdere, hvordan min adfærd påvirker min sundhed.

9	I can usually figure out if some health-related information is right or wrong	Jeg kan som regel finde ud af, om information om sundhed er rigtig eller forkert.

10	I can give reasons for choices I make regarding my health	Jeg kan forklare de valg jeg træffer i forhold til min sundhed.

In the third phase, 82 schools in 3 of the 5 regions in Denmark were invited to participate in a study on children's food literacy, which also included the final HLSAC instrument. Fifteen schools (10 public and 5 private) agreed to participate. Data were collected from pupils in grades 6 to 7 using electronic questionnaires during single lessons from March 2019 to April 2019.

## Statistical Analysis

The original HLSAC was developed based on five factors; however, the five-factor model had problems with high correlations between factors, and instead a one-factor model was suggested, tested, and deemed acceptable (Paakkari et al., 2016). Therefore, we chose the one-factor model.

The structural validity of the HLSAC instrument was assessed using confirmatory factor analysis (CFA) with the RStudio Version 1.0.153 and Package lavaan version 0.6–3. The ordinal nature of the items was accounted for in the model. The model fit was assessed using commonly used goodness-of-fit indices. The following criteria indicating excellent model fit were used: comparative fit index (CFI) >0.95, Tucker-Lewis Index (TLI) >0.95, standardized root mean square residual (SRMR) <0.08, root mean square error of approximation (RMSEA) <0.06, and df/chi2 <5 (Hu & Bentler, 1999).

For skewness we used the criterion suggested by Kim (2013) that for samples exceeding 300, skewness should be between −2 and 2.

The predictive validity of the instrument was tested using regression analysis with food intake as a health behavior outcome of HL. Food intake was selected as an example among outcomes used in other HL studies such as physical activity, smoking, and substance use (Klinker et al., 2020; Paakkari, Torppa, Paakkari, et al., 2019). A food frequency index was calculated by summing scores from five frequency questions on the intake of fruit, vegetables, fish, sweets or chocolate, and soft drinks containing sugar (Rasmussen et al., 2015).

The internal consistency of the instrument was assessed using Cronbach's alpha. Values >0.7 were considered acceptable (Ponterotto & Ruckdeschel, 2007).

## Ethics

Informed parental consent was achieved prior to data collection. The pupils were informed on the day of data collection that their participation was voluntary, only the research team would see their responses, and only group-level results would be published. The study was approved by the Capital Region Denmark (ref. no. VD-2019-107) and adhered to General Data Protection Regulation (GDPR) regulations.

## Results

A total of 1,040 pupils in grades 6 to 7 were invited to participate, and 805 (77%) completed the questionnaire. No pupils with parental consent declined to participate. The pupils' mean age was 12.2 years.

The mean item-specific HL scores ranged from 2.79 (item 3) to 3.31 (item 4). The response distribution indicated a ceiling effect for all 10 items, as 15% to 44% of the pupils chose the highest response category. Item 4 and 10 had the highest absolute skewness values (−.77 and −.57); however, no items had problematic skewness (**Table 1**). The mean HL scores did not differ by grade (*p* = .210) or school type (*p* = .666). There was a borderline significant difference by gender (*p* = .055) (**Table 2**).

**Table 2:**
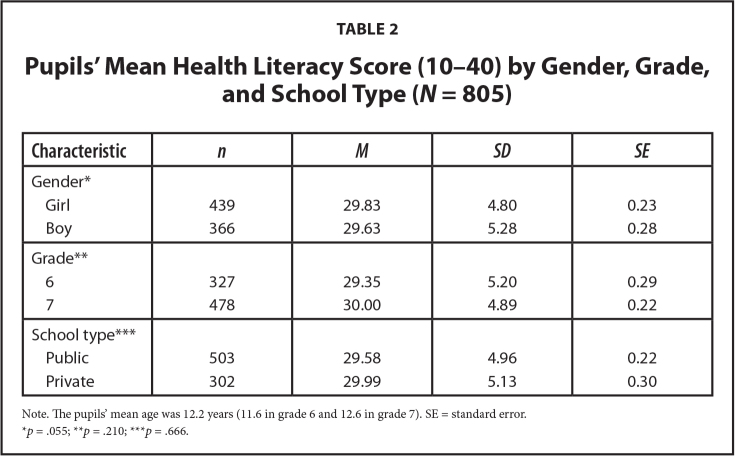
Pupils' Mean Health Literacy Score (10–40) by Gender, Grade, and School Type (*N* = 805)

**Characteristic**	***n***	***M***	***SD***	***SE***

Gender^*^				
Girl	439	29.83	4.80	0.23
Boy	366	29.63	5.28	0.28

Grade^**^				
6	327	29.35	5.20	0.29
7	478	30.00	4.89	0.22

School type^***^				
Public	503	29.58	4.96	0.22
Private	302	29.99	5.13	0.30

Note. The pupils' mean age was 12.2 years (11.6 in grade 6 and 12.6 in grade 7). SE = standard error.

* *p* = .055;

** *p* = .210;

*** *p* = .666.

When HL was modeled as one latent factor with all 10 items loading on this factor, the CFA showed standardized factor loadings ranging from 0.52 to 0.75, (*p* < .001). The fit indices indicated an excellent fit (*df* = 35, chi^2^ = 79.896, df/chi^2^ = 0.4); C*FI* = 0.99, T*LI* = 0.99, SR*MR* = 0.038, RMS*EA* = 0.040, confidence interval: 0.028–0.052).

The regression analysis revealed that HL was significantly associated with food intake (*p* < .001, r^2^ = 0.027), explaining 2.7% of the variance. Further, the instrument showed high internal consistency (Cronbach's alpha = 0.86).

## Discussion

After translation and validation, the data from a convenience sample of 805 pupils in grades 6 to 7 showed an excellent model fit related to the structural validity of the HLSAC instrument and high internal consistency.

The meticulous translation process is a study quality, and the high internal consistency is a clear strength of the instrument. The predictive ability of the instrument related to healthy food intake indicates its promising utility, even if it only accounted for 2.7% of the variance in food intake. Food intake is just one of many behavioral outcomes of HL, and a small or medium variance is expected in this kind of research (Abelson, 1985). A larger Finnish study using the HLSAC instrument found that HL explained eight health behaviour measures, including healthy food intake (Paakkari, Torppa, Paakkari, et al., 2019). The association of HL with healthy food behaviour was recently also shown among Danish vocational students using another HL instrument (Klinker et al., 2020).

Compared to the original HLSAC instrument, the Danish version showed better model fit indices. One possible explanation could be that we adjusted for the categorical nature of the variable, which was not done originally.

HL was modeled in a one-factor model, although the instrument is based on five theoretical components, and it is possible in such a case to use a second-order factor model. There are pros and cons of this issue, as capturing the complexity of the HL construct by defining subscales increases the “face validity” of a HL scale but violates the assumption of a unidimensional interval scale and, hence, the requirement of additivity (Finbråten et al., 2017). Given this explicitly multidimensional design, research has warned that most HL scales have a multidimensional structure, which implicitly suggests that we should question the plausibility of claims about people's HL based on the sum score of composite HL scales (Altin et al., 2014).

## Limitations

Eight of 10 items exhibited symmetrical distribution, but the remaining two items had skewness values < −0.5, but > −1, which indicates moderate skewness. The responses to all 10 items exhibited a ceiling effect, defined as >15% of responses in the highest response category, which is a limitation for the instrument's potential use to measure progress in a pre-post evaluation. A recent study translated and validated a HL questionnaire in six Asian countries and found neither a floor nor a ceiling effect (Duong et al., 2017), whereas the original instrument exhibited similar ceiling effects as our translated version (Paakkari, Torppa, Villberg, et al., 2018).

Our study was conducted in a convenience sample, which is considered acceptable for a validation study, but not for studies aiming to provide representative data on HL levels. Further, our study population was pupils in grades 6 to 7 (age 11–14 years) in contrast to the Finnish study (Paakkari, Torppa, Villberg, et al., 2018) that included a representative sample of 3,833 pupils in grades 7 and 9 (ages 13 and 15 years). We found no difference by grade, indicating the instrument's potential usefulness in younger ages. The Finnish study found higher HL in older pupils. We found a borderline significantly higher HL in girls compared to boys, which is in line with the Finnish results. Further studies in Denmark should be conducted on a representative sample and include older children to confirm these findings. Future research should also investigate other types of validity (e.g., discriminatory) and reliability (e.g., test-retest).

## Conclusion

The findings suggest that the Danish version of the 10-item HLSAC instrument is a reliable and valid instrument for measuring HL in children and adolescents age 11 to 14 years. The instrument is ready to use in larger representative surveys in Denmark to monitor prevalence of HL, guide health promotion, and provide data for further exploration of the potentials and limitations of the instrument.
